# Dietary Patterns, Occupational Stressors and Body Composition of Hospital Workers: A Longitudinal Study Comparing before and during the COVID-19 Pandemic

**DOI:** 10.3390/ijerph20032166

**Published:** 2023-01-25

**Authors:** Carlos Rodrigo Nascimento de Lira, Rita de Cássia Coelho de Almeida Akutsu, Lorene Gonçalves Coelho, Renata Puppin Zandonadi, Priscila Ribas de Farias Costa

**Affiliations:** 1 School of Nutrition, Federal University of Bahia, Avenida Araújo Pinho, n°32, Canela, Salvador CEP 40110-150, Brazil; 2 Department of Nutrition, Campus Darcy Ribeiro, University of Brasilia, Asa Norte, Distrito Federal, Brasília CEP 70910-900, Brazil; 3 Health Science Centre, Federal University of Recôncavo of Bahia, Avenida Carlos Amaral, n°1015, Cajueiro, Santo Antônio de Jesus CEP 44430-622, Brazil

**Keywords:** occupational stress, food pattern, body composition, COVID-19 pandemic

## Abstract

This longitudinal study aimed to evaluate the association between dietary patterns and the body composition of hospital workers subjected to occupational stressors before and during the COVID-19 pandemic. Data on sociodemographic, occupational, lifestyle, anthropometric, food consumption and occupational stress were collected before and during the COVID-19 pandemic. A total of 218 workers from a private hospital in Santo Antônio de Jesus, Bahia, Brazil were included in the study. After evaluating the normality of the data, parametric or non-parametric tests were used to characterize the sample. Dietary pattern was defined with Exploratory Factor Analysis and Structural Equation Modeling was used to test the desired association. During the pandemic, work per shift increased by 8.2% (*p* = 0.004) and working hours > 40 h/week increased by 9.2% (*p* = 0.006). Despite the higher prevalence of low occupational stress (85.8% vs. 72.1%), high stress increased by 13.7% from 2019 to 2020 (*p* < 0.001) and 30.3% reported a positive mediating effect on the variables of body composition, body mass index (b = 0.478; *p* < 0.001), waist circumference (b = 0.395; *p* = 0.001), fat-free mass (b = 0.440; *p* = 0.001) and fat mass (b = −0.104; *p* = 0.292). Therefore, a dietary pattern containing high-calorie foods was associated with changes in the body composition of hospital workers, including occupational stressors as mediators of this relationship.

## 1. Introduction

Work and health are two sides of the same coin, or are still considered as intrinsic life factors [1]. If, on the one hand, work serves to dignify the individual as a being who lives in society, belonging to a system of rights and duties, on the other hand, it can be favorable towards deleterious health outcomes [2,3,4,5,6,7]. In this scenario, there is a consensus that the hospital is one of the most unsafe work environments, leaving workers at constant biological risk (e.g., infections and contagious diseases) and/or non-biological risks (e.g., occupational stress) [8,9].

Among the outcomes related to the workers’ health within these complex workspace dynamics, body composition is an important factor for attention, as it is a vital component of an individual’s health and results from the interaction between genetic and environmental factors [10]. The excess or lack of food is harmful to human beings and an important factor in defining an individual’s body composition. However, body composition does not result exclusively from food intake, given that food is not categorized as good or bad, not even between those able to increase weight. Both the dietary pattern and stressors at work are environmental factors that contribute to the complexity of body composition. Thus, the assessment of dietary patterns is a more appropriate alternative to food ingestion evaluation, as it does not consider only the nutrients ingested but also the cultural, social, economic and demographic factors, revealing that eating is complex and dynamic [11], and reflects on how different foods (and nutrients) are combined in an individual’s usual diet [12,13].

In this study, the dietary pattern is understood as a set of foods consumed, identified from the correlation between them in a given population [14,15]. Investigations on this topic in hospital workers are limited, with most studies focusing on food consumption or eating behavior [16,17,18]. Hospitals are places where occupational stressors are part of professionals’ daily lives [19,20,21,22,23]. In addition, the global crisis triggered by the COVID-19 pandemic not only sharpened the structurally present occupational stressors, but also produced new stressors, which added to the pre-existing ones [24]. Studies pointed out working stressors influencing these professionals’ eating habits and nutritional status, as shift work is associated with a lower protein and higher energy, carbohydrates and lipids intake; skipping meals is associated with long working hours; and a higher frequency of eating in snack bars [25,26,27]. Therefore, excess weight was not just a result of personal choices [23,28,29,30], particularly during the COVID-19 pandemic in which the physical and mental exhaustion that was exacerbated in this period caused an imbalance in energy intake and an excessive use of oil and salt in meals [31] and forced health professionals to eat at non-habitual times [32]. Additionally, carbonated and sweetened beverages, which are very common in the Western dietary pattern, contribute to an increase in caloric intake and a consequent increase in the values of body composition parameters and in the prevalence of chronic diseases [12,33].

It is known that hospital workers, especially health professionals, are highly exposed to occupational stressors and have a high prevalence of excess weight, often due to work stress. At the same time, their health is essential, reflecting their productivity, patient safety and well-being [34,35,36,37] and absenteeism reduction [34]. To date, no studies were observed in the consulted literature referring to the effects of dietary patterns on body composition, with the possible role of occupational stressors in this relationship, for this population using Structural Equation Modeling (SEM). Therefore, this study aimed to investigate the relationships between dietary patterns, body composition and occupational stressors among hospital workers before and during the COVID-19 pandemic.

## 2. Materials and Methods

This is a prospective cohort study carried out with workers from a medium-sized private hospital [35], in the municipality of Santo Antônio de Jesus, Bahia, Brazil. This study is part of a larger project evaluated and approved by the Ethics and Research Committee of the School of Nutrition at the Federal University of Bahia, with due updates (CAAE approval number 4,316,252).

### 2.1. Sample and Eligibility Criteria

We used a convenience sample in which all 371 professionals working in the hospital’s staff in 2019 (regardless of the work sector, e.g., emergency, outpatient clinic, nutrition, nursing, pharmacy, hygiene, administrative) were invited to participate in the study and were evaluated on the eligibility criteria. The inclusion criteria were: working at the hospital; and ≥18 years old (age of majority in Brazil). Workers with problems that compromised the performance of anthropometry, individuals in a postoperative period of surgery to the abdomen, pregnant women, or women in the puerperium (last six months), due to changes in body composition in this phase, were determined as the life-related exclusion criteria [36]. Another exclusion criterion was questionnaires incorrectly or incompletely completed. However, the researchers asked the workers for all the information of interest, so there was no incorrect or incomplete completion of questionnaires. For this reason, no questionnaires were excluded.

Of 371 workers, 69 did not accept, 66 were dismissed from work during the study, 8 were on medical leave, 4 were pregnant women (meeting the exclusion criteria) and 4 were on vacation during the data collection. Thus, the sample was composed of 218 workers who consented to their participation.

**Figure 1:**
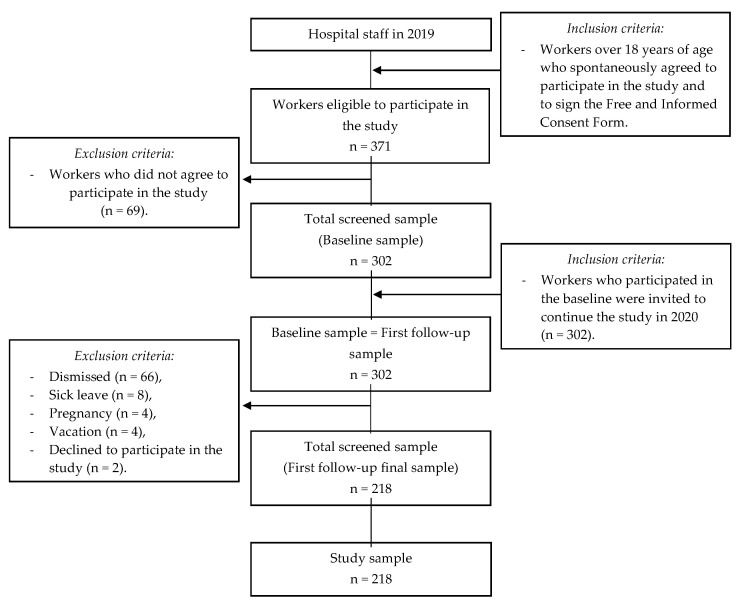
Graph demonstrating the process of obtaining the sample. Coelho, L.; Costa, P.; Kinra, S.; Mallinson, P.; Akutsu, R. Association between occupational stress, work shift and health outcomes in hospital workers of the Recôncavo of Bahia, Brazil: The impact of COVID-19 pandemic. *British Journal of Nutrition,* 2023, *129*(1), 147-156, reproduced with permission [39].

The sample has a power of 87%, 82% and 84% to identify prevalences of overweight, obesity and abdominal obesity in individuals in the highest tertiles of consumption of a Western diet, a healthy diet, animal meat and alcoholic beverage consumption patterns, respectively, considering the proportions of 29.5%, 42.3% and 41% overweight; 37%, 40.7% and 33.3% of obesity; and 40.7%, 39.5% and 33.7% of abdominal obesity in Western, healthy and animal meat and alcoholic beverage standards, respectively [37]. The calculations of the sample power (1-ß) were based on a significance level of 5%, two measures repeated in time and two-tailed tests, indicating that this sample size was sufficient to carry out unbiased estimates of the population parameters under study [38]. The process for obtaining the sample is detailed in Figure 1.

### 2.2. Data Collection

With the hospital’s and the workers’ consent, in 2019 the data were collected by a team previously trained in the research protocol, and under the supervision of the responsible researchers, to compose the baseline data, and after a minimum interval of 12 months, configuring the survey follow-up. The information collected was: sociodemographic (gender, age, self-reported skin color, schooling and family income), occupational (profession, work position, length of time working in the position, work shift, working hours, the workload of weekly work, type of employment contract and presence of another job) and perception of health (perception of own health and self-report of diagnosis of COVID-19).

As for lifestyle, the self-reported variables of interest were smoking, alcoholism, hours of sleep and level of physical activity. The physical activity was assessed using the reduced version of the International Physical Activity Questionnaire (IPAQ), and workers classified as low (<600 Metabolic Equivalents (MET)—minutes/week), moderate (600–3000 MET—minutes/week) and high level of physical activity (≥3000 MET—minutes/week) [40].

Anthropometry was performed by measuring weight (portable digital scale with bioimpedance on a platform) and height (measured with a portable stadiometer), using techniques established by the World Health Organization (WHO) [41,42]. With such information, the Body Mass Index (BMI) was obtained [6] and classified according to the WHO proposition [42]. Waist circumference (WC) was measured with a flexible and inelastic measuring tape, following WHO recommendations [41]. With WC values, it was possible to predict the risk of metabolic and cardiovascular complications of workers from the cutoff points proposed by the WHO [43], for women (WC < 80 cm without risk, WC of 80–88 cm increased risk and WC > 88 cm greatly increased risk) and for men (WC < 94 cm without risk, WC 94–102 cm increased risk and WC > 102 cm greatly increased risk). The measurements were taken at the same time and under the same conditions, as a specific space was available for data collection.

Body composition was measured using a tetrapolar bioelectric impedance device (Biodynamics^®^), according to the protocol described by Kyle et al. [44]. The classification of the workers’ body fat percentage was based on the parameters proposed by Guedes and Guedes [45], which considered 15–19.99% light, 20–24.99% moderate, 25–29.99 high and ≥30 % morbid for males and 25–29.99% mild, 30–34.99% moderate, 35–39.99% severe and ≥40% morbid for females.

The Food Frequency Questionnaire (FFQ) of the Longitudinal Study of Adult Health—ELSA—Brazil [46] was used to obtain dietary information. This FFQ contains a list of foods made up of 114 items, structured in three sections: (1) foods/preparations, (2) measurements of consumption portions and (3) frequency of consumption, with eight categories: “more than 3 times/day”, “2–3 times/day”, “1 time/day”, “5–6 times/week”, “2–4 times/week”, “1 time/week”, “1–3 times/month” and “never/almost never”. The instrument has satisfactory reliability for all nutrients and reasonable relative validity for energy, macronutrients, calcium, potassium and vitamins E and C [46]. The dietary food patterns that were categorized as “Pattern A” consisted mainly of traditional foods rich in micronutrients such as tubers, fruits, vegetables, oilseeds and eggs; “Pattern B” was represented by foods such as meat, pasta, and other dishes, sweets and sugary drinks; and “Pattern C” consisting of foods such as bread and cereals, milk and dairy products, fats and beverages.

Finally, levels of occupational stress were obtained using the reduced version, translated and validated for the Brazilian population, of the Job Content Questionnaire (JCQ) [47]. This questionnaire has an internal consistency (Cronbach’s alpha coefficients) of 0.72 for demand, 0.63 for control and 0.86 for social support. From the Demand-Control Model, the responses for demand and control were summed and the median was calculated. Then, the participants were classified into one of the four quadrants of the model: (1) high strain, when there was a value above the median for demand and below the median for control; (2) low wear, if above the median for control and below the median for demand; (3) passive when both demand and control were below the median; (4) active, when both domains were above the median. Finally, workers were categorized into high occupational stress if they had high strain; and in low occupational stress if they presented as low wear, passive work and active work.

### 2.3. Statistical Analysis

To characterize the sample at baseline (2019) and follow-up (2020), we performed simple (*n*) and absolute (%) frequencies for categorical variables, the mean and standard deviation for continuous quantitative variables, which had their normality assessed by Shapiro–Wilk. The difference between the periods (before and during the pandemic) was evaluated using Pearson’s Chi-square, McNemar, Wilcoxon Test or Student’s *t*-test, depending on the variable type. We adopted a significance of 5% (*p* ≤ 0.05) between analyses.

Given the state of the public health calamity instituted by the COVID-19 pandemic and its consequent impacts on the health of hospital workers [18,48], we decided in this study to use the follow-up information (when the pandemic was at its critical moment in Brazil) for the multivariate analysis of the data. In this sense, the workers’ dietary patterns were identified using an a posteriori approach [14] based on exploratory factor analysis by principal component. The Kaiser–Meyer–Olkin (KMO) and Bartlett’s sphericity tests were used to assess the adequacy of the data for the analysis, considering acceptable values above 0.60 and *p* < 0.05, respectively [14,49]. The commonality, that is, the extent to which there is a connection between the group (variable) and the factor (standard), was acceptable if >0.30 [14,49]. The number of factors to be retained was defined by the eigenvalues criterion or the Kaiser criterion (>1 as a cutoff point) [50]. The Varimax orthogonal rotation of the factors was used to more effectively interpret the data. A factorial load > 0.30 was adopted as a selection criterion for the food groups to be included in the pattern [14,49].

To evaluate the theoretical model established about the association between dietary patterns and body composition mediated by occupational stressors, we used the analysis technique of Structural Equation Modeling [49]. The primary exposure variable (exogenous variable) was the dietary pattern and included in the model as a continuous factorial score. The indicator variables of body composition BMI, WC, body fat and fat-free mass formed the outcome variable (endogenous), and were included in the continuous form. The observable variables COVID-19, workday, work shift, and occupational stress (represented by demand and control) in categorized form, originated the latent variable occupational stressors. Thus, the latent constructs (measurement models) were measured [51]. To evaluate the structural model constituted by the observed and latent variables, unadjusted and standardized regression coefficients were estimated, with 95% confidence intervals (95%CI) and *p*-value, the direct and indirect effects of the model, as well as the model fit indices.

The estimation method adopted was the Diagonally Weighted Least Squares (DWLS), which is suitable because it produces a more reliable model inference with small to medium sample sizes and is more likely to detect small structural relationships when there are categorical data in the model, and the data are slightly or moderately asymmetric [52]. Finally, the final model chosen was based on the adequacy of the adjustment indices, ensuring the validity, significance and plausibility of the estimates obtained by the tested models. The residual fit indices adopted were the Root Mean Square Error of Approximation (RMSEA < 0.06) [53] and the Standardized Root Mean Square Residual (SRMR < 0.08) [49], whereas the comparative indices were the Tucker–Lewis Index (TLI ≥ 0.90) [49] and the Comparative Fit Index (CFI ≥ 0.90) [54].

The Statistical Package for Social Sciences software, version 21.0, was used for data entry and descriptive and exploratory factor analysis to determine dietary patterns. Jamovi software, version 2.2.5.0, was used for Structural Equation Modeling analysis.

## 3. Results

### 3.1. Characteristics of Hospital Workers

The hospital workers who composed the sample of this study were mostly women (75.2%), with complete secondary education (50.2%), married (52.3%), who worked in the hospital’s administrative sector (58.3%), where the employment contract was formal (95%), that is, following Brazilian labor laws and workers had been working in hospitals for more than 12 years (77.1%) (Table 1).

Regarding the characteristics of these workers before and during the COVID-19 pandemic, we observed that in both periods, the income was from one up to three minimum wage (*p* < 0.05); alcoholism increased by 8.3% (*p* = 0.004); there was a decrease in the number of workers who slept < 7 h/day (*p* = 0.043); the number of workers who had a day shift decreased by 7.8%. On the other hand, those who started to work on duty increased by 8.2%, and such changes were statistically significant (*p* = 0.004). Working hours > 40 h/week increased by 9.2% (*p* = 0.006) (Table 2).

Although in this sample there is a higher prevalence of low occupational stress (85.8% vs. 72.1%) among workers, high stress increased significantly (*p* ≤ 0.001) by 13.7% from 2019 to 2020. Additionally, 30.3% of respondents reported a positive diagnosis of COVID-19 (Table 2).

Significantly higher values of body weight (*p* < 0.001), waist circumference (*p* < 0.001) and body fat percentage (*p* < 0.001) were found during the COVID-19 pandemic compared to 2019. However, there was a reduction of 1.4 in mean fat-free mass (*p* < 0.001) (Table 2).

### 3.2. Dietary Pattern of Hospital Workers

Three dietary patterns were retained in the assessment during the COVID-19 pandemic. Together they represented 44.47% of the total variance. All food groups selected for each pattern showed significant correlations with the component (r ≥ 0.30) (Table 3).

The “Pattern A” accounted for 17.20% of the total variance, “Pattern B” corresponded to 14.71% of the total variance and “Pattern C” presented a variance of 12.56% (Table 3).

### 3.3. Food Pattern and Body Composition of Hospital Workers

**Figure 2:**
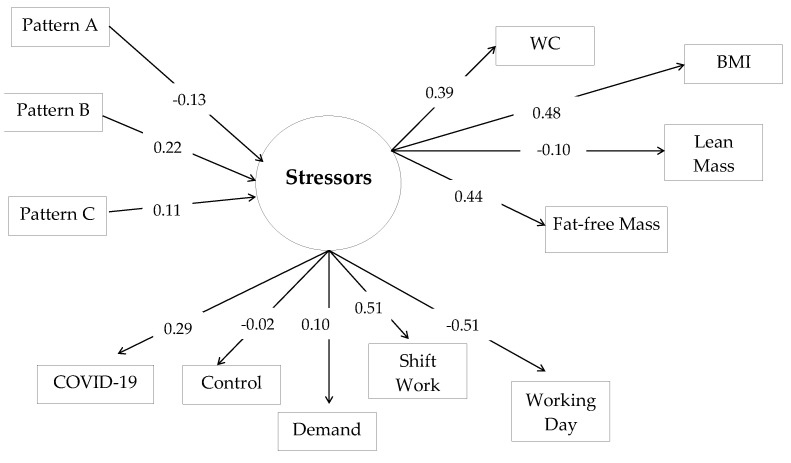
Modeling of Structural Equations for associations between dietary pattern, occupational stressors and body composition in hospital workers. BMI = Body Mass Index; WC = waist circumference.

The three dietary patterns directly affected the latent variable occupational stressors, and only in Pattern A (b = −0.133; *p* = 0.185) was this effect negative. In Patterns B (b = 0.225; *p* = 0.023) and C (b = 0.144; *p* = 0.278) there was a direct and positive effect on the latent variable, but Pattern B was the only statistically significant one. Still, the latent variable occupational stressors had a direct and positive mediating effect on the variables of body composition, BMI (b = 0.478; *p* < 0.001), WC (b = 0.395; *p* = 0.001), fat-free mass (b = 0.440; *p* = 0.001) and fat mass (b= −0.104; *p* = 0.292), and this effect was only not statistically significant for the association with fat mass (Table 4 and Figure 2).

The results of the standardized estimates indicated a positive and significant indirect effect of Pattern B on the variables BMI (b = 0.107; *p* = 0.025), WC (b = 0.089; *p* = 0.027) and fat-free mass (b = 0.099; *p* = 0.032), which referred to body composition (Table 4). The fit indices showed a good fit for the model.

## 4. Discussion

The results of this study made it possible to identify that dietary pattern B (made up of meat, pasta and other preparations, sweets and sugary drinks), was associated with hospital workers’ body composition indicators mediated by occupational stressors. Pattern B (b = 0.225; *p* = 0.023) was also directly associated with the mediator variable occupational stressors, associated with BMI, WC and fat mass, three of the four indicators of body composition defined as outcomes in this study. Thus, exposure to dietary patterns impacts body composition when mediated by occupational stressors.

Fit indices, which inform how well a model can reduce the raw data, or even that the theoretical model fits well to the sample data [49], showed that the proposed model is appropriate. The RMSEA, in addition to showing a value considered good (<0.05) [53], showed a narrow confidence interval (95%CI: 0.000–0.054) and a non-significant *p*-value (*p* = 0.913), reinforcing that there was no significant discrepancy between the created model and the covariance structure of the data [53].

Among the eating patterns identified in this study, pattern A and pattern C did not show a statistical significance for the relationship tested, which can be explained by the types of foods that represented such patterns. Foods that formed food pattern A (tubers, fruits, vegetables, oilseeds and eggs) are consistently considered healthy, therefore, not interfering with weight gain. Food pattern C (bread and cereals, fats, milk and dairy products, beverages) was represented by foods that, based on well-established choices made by individuals, are not foods capable of impacting body weight. Such findings are consistent with other studies carried out with health professionals in Iran [37], Brazil [11] and Mexico [12].

Dietary pattern B had a statistically significant effect on the latent variable occupational stressors. The foods that compose this dietary pattern are characterized by a high energy value, lipids and simple carbohydrates. Therefore, their frequency and amount consumed influence body composition [55].

In the neuroendocrine system, the components involved with obesity are the afferent system, the processing unit of the central nervous system, and the efferent system [56]. Despite the complexity of the factors for determining the outcome obesity, low levels of physical activity and high food consumption are environmental factors that determine such an outcome. In a context of stress, such as at work, individuals tend to have a high food intake, mostly hyper-palatable foods with a high caloric density [57], favoring an increase in BMI and fat mass [55].

Physiologically, weight gain as a result of work dynamics is represented by increased stress, which acts on the activation of the hypothalamic–pituitary–adrenal axis, resulting in increased cortisol and metabolic changes, leading to increased body fat storage [30]. Additionally, the psychosocial aspects and demands of the work environment can favor the reduction in physical activity and hours of sleep (or poor quality of sleep), and promote the consumption of caloric and nutrient-poor foods, contributing to weight gain [58,59,60].

Shift work is the leading occupational stressor investigated and associated with the lifestyle of hospital workers. Shift work is involved in the professional’s sleep deprivation and, in hospitals (where work is uninterrupted), the damage is unquestionable and seems to increase the risk of visceral obesity [61,62,63]. This work condition is also responsible for shaping the workers’ eating habits compared to those who do not work in shifts, showing a greater variability in daily caloric intake; greater consumption of caloric snacks [64]; irregular meals during work [26], among other behaviors that contribute to increased WC, BMI and the risk of metabolic syndrome [65]. A meta-analysis [66] concluded that factors such as circadian misalignment, meal times, food choice and variation in energy metabolism at night might be responsible for the increased rates of obesity observed in shift workers.

In dietary pattern B, the highest factorial load was observed in the Pasta and Other Preparations group (0.766), represented by foods/preparations such as pizza, pasta, instant noodles, fried snacks, Bahian food (typical regional food, whose ingredients are okra, palm oil, salted and dried shrimp, cashew nuts and roasted peanuts), Japanese food and industrialized soups, among others. The Sugary Beverages group (0.654) was responsible for the second highest factorial load and was represented by soda, industrialized juice and tea, sweetened coffee, etc. The Sweets category with the lowest factorial load (0.390), were ice cream, caramel, gelatin, chocolate powder, chocolate and pudding, among others.

Carbohydrates show digestion and absorption rates that affect postprandial glycemic and insulinemic responses [30]. However, factors such as the food’s physical form, chemical composition and processing type, among others, will determine this food’s speed of digestion, absorption and glycemic index [30]. Among the carbohydrate sources used in preparations, sweets and sugary drinks have a higher glycemic index. After carbohydrate consumption, there is greater insulin secretion, favoring the rapid absorption of glucose in the cells, thus increasing glucose oxidation and reducing lipid oxidation. Thus, this process results in excess weight and increased body fat, especially visceral adiposity [67,68].

The dietary pattern shown by our study corroborates the food consumption of hospital workers in other parts of the world. In South Africa, Kunene and Taukobong [16] evaluated 109 health professionals and showed high percentages of consumption of sweet foods (60%), sweetened beverages (55%), coffee (64%) and alcohol (65%), unhealthy snacks (50%), salty snacks (36%), fast food (37%), fatty foods (38%), fried foods (49%) and foods with a lot of sugar (47%). In Saudi Arabia, Al Hazmi, Alghamdi and Abdulmajeed [69] evaluated the eating habits of 388 hospital health professionals. The foods most consumed by them during working hours were sweets (46.6%), cereals and bread (42.8%), meat/fish/eggs (39.9%), coffee (66.2%) and tea (35.8%). In Ghana, Nuhu, Ainuson-Quampah and Brown [17] observed that the daily consumption of food by nurses in hospitals was less frequent for fruit juice (7.1%), whereas in weekly consumption, soft drinks were the most consumed (32%).

The sources of lipids identified in dietary pattern B are mainly from preparations such as Bahian food (which contains palm oil) and fried and/or baked snacks. Despite being from a vegetable source, palm oil is composed of saturated fatty acids (45% palmitic acid and 5% stearic acid) and unsaturated acids (40% oleic acid and 10% linoleic acid) [70] and seems to behave similarly to fats of an animal origin concerning plasma lipids [71,72]. Considering that adipose tissue stores saturated fatty acids more efficiently, their consumption should be cautious following the nutritional recommendation for maintaining good cardiovascular health [70].

In this study, the Meat group, which also integrated dietary pattern B, was responsible for the third-highest factorial load (0.607). It was represented by beef liver, beef, pork, roasted or cooked chicken cuts, fried or boiled fish, seafood, bacon and processed meats such as hamburgers and sausages. Nutritionally, despite being a rich source of protein of high biological value, meat also contains fatty acids, which vary according to the animal and the type of cut, with monounsaturated and saturated fatty acids being the most common [70]. In a Ghanaian study, daily food consumption reported by nurses was also higher for animal protein (89.4%) [17].

Brazil is a great producer and consumer of meat globally [73]. The high intake of red and processed meat caused an abrupt increase in diet-related non-transmissible chronic diseases [73]. In addition, its high consumption is also associated with increased cardiovascular risk, reasons why a moderate intake is recommended and according to the total saturated fatty acids recommended in the diet [70]. In addition, because it is nutritionally considered a food with a high energy density (due to its fat content), its consumption may also be related to higher weight values and, consequently, BMI and WC [74].

Although stress is a part of human life, precariousness in work has favored the occurrence of occupational stress, frequently representing an important risk factor for many diseases. In occupational stress, negative impacts on health, quality of life, the performance of organizations and reduction in work capacity can be observed [75].

Aiming to determine the number of latent BMI trajectories among Canadian workers over 17 years, and to determine how the psychosocial and physical trajectories of the work environment were associated with latent BMI trajectories, Dobson et al. [76] found four distinct trajectories of BMI (normal weight, overweight, obesity and very obese) among workers and that all groups had an increase in BMI of at least 1.62 kg/m^2^ throughout the study. Lower decision-making authority and decreased physical exertion were associated with belonging to the overweight and obesity trajectories. Therefore, the authors reinforce that controlling the body weight of workers is important to their health maintenance.

Studies suggest that occupational stressors impact on eating behaviors, leading to excess weight [77,78], while others do not demonstrate this association [79,80]. Shafi, Arif and Nasir [27] observed that among 300 Pakistani nurses, 61% worked long hours and that this working condition was associated with BMI (*p* < 0.05) and long working hours were associated with missing meals (*p* ˂ 0.05). In Finland, a cohort [81] identified that, among men who worked for an airline, increased levels of occupational stress were associated with increased fat consumption (β = 0.59, 95%CI: 0.07–1.11) and saturated fat (β = 0.31, 95%CI: 0.02–0.58).

In our study, occupational stressors were often associated with the hospital environment (work shift, journey and high occupational stress) increased between 2019 and 2020, indicating that they were heightened by the COVID-19 pandemic. Thus, this pandemic has become another stressor in the work environment, especially among hospital professionals, given they are at greater risk of SARS-CoV-2 infection when in close contact with infected people [82]. Although most of our sample reported not having been infected with the virus during the study, the disease impacts worsened their working conditions [18,48,83].

Regarding eating habits, this pandemic was responsible for the imbalance in energy intake and the excess of oil and salt in the meals provided to health professionals and was responsible for the change in body weight, even in a short period of months [31]. Among 403 health professionals from hospitals in Turkey, the frequency of meals and the consumption of canned food, fruits, soft drinks, tea/coffee and red meat, among others, showed a statistically significant difference before and during the COVID-19 pandemic (*p* < 0.05). Corroborating our findings, the authors identified that the frequency of eating frozen and canned foods, fruits, nuts, tea/coffee and red meat, among others, increased during the crisis [18]. In a Brazilian study, among 710 health professionals interviewed, 78.5% said they had changed their diet during pandemic, represented by increased carbohydrate intake and night-time food intake alone. In addition, 27% of individuals reported increased consumption of alcoholic beverages, mainly wine (14.2%) and beer (11.2%) [84].

In summary, the relationship between work and obesity can, for example, be due to the characteristics of the work, the eating environment at work, the presence of occupational stress, rotating/night work, etc. [85]. Therefore, in the presence of occupational stressors, eating habits and physical activity patterns are compromised [86,87,88], or moreover, through an indirect effect, through income, which can affect the acquisition of a healthy diet [89]. With this, it is evident that taking care of the diet of these professionals, especially during a period of response with intense work and physical and mental exhaustion, is extremely necessary. In addition, considering the epidemiological and clinical importance of occupational stressors for measuring workers’ body composition, monitoring food consumption and anthropometric measurements and clinical assessment strategies to be adopted in medical services focused on workers’ health are necessary.

As a limitation of this study, there is the fact that the sample consisted of only one hospital. Therefore, caution should be taken when generalizing the results beyond the study. However, we reinforce that, with the pandemic, it was not possible to access many hospital units. Second, as information on food consumption was collected using a food frequency questionnaire, there is a risk of information bias. Finally, in this study, we did not evaluate the use of drugs that can alter the regulation of hormones, which may be involved in increasing anthropometric parameters. As strong points, we highlight the evaluation of the dietary pattern in this population through a robust methodology; reliability in obtaining the data, with qualified interviewers who checked the records for any incorrect or missing information; assessment of body composition using anthropometry and bioimpedance, which improves the relevance of results.

## 5. Conclusions

The results of this study reveal that a dietary pattern containing calorie-dense foods favors changes in the body composition of hospital workers, including occupational stressors as mediators of this relationship. Thus, the findings suggest the need for greater attention to nutritional care in the work environment, particularly in times of crisis, such as the COVID-19 pandemic, since this outcome poses a risk for chronic non-communicable diseases, metabolic syndrome and other morbidities associated with body pattern. In addition, our discoveries contribute to the guidance, direction and strengthening of food and nutrition policies in the context of workers’ health, which includes changes in eating habits, in the ways of preparation and the acquisition of food considered healthy.

## Figures and Tables

**Table 1:** Characteristics of hospital workers at baseline. Santo Antônio de Jesus, Bahia, Brazil, 2019.

Variable	Category	*n*	%
Sex	Men	54	24.8
	Women	164	75.2
Educational level	Primary school	09	4.1
	High school	110	50.5
	College/university education	75	34.4
	Postgraduate education	24	11.0
Skin color	White	28	12.8
	Brown	106	48.6
	Others	84	38.5
Marital status	Married	114	52.3
	Single	92	42.2
	Others	12	5.5
Occupation	Administrative	127	58.3
	Health professional	91	41.7
Employment contract	Effective (CLL)	207	95.0
	Others	11	5.1
Time in office	≤12 months	50	22.9
	>12 months	168	77.1

CLL = Consolidation of Labor Laws.

**Table 2:** Characteristics of workers before and during the COVID-19 pandemic. Saint Anthony of Jesus. Bahia Brazil.

**Categorical Variables**	**Category**	**Before (2019)**		**During (2020)**		***p*-Value #**
		***n***	**%**	***n***	**%**	
Family income *	>7 minimum wages	14	6.4	10	4.6	0.000
	<1 minimum wage	16	7.3	02	0.9	
	1 to <3 minimum wages	130	59.6	133	61.0	
	3 to 5 minimum wages	47	21.6	56	25.7	
	5 to <7 minimum wages	11	5.0	17	7.8	
Smoking	Yes	02	0.9	04	1.8	0.098
	No	216	99.1	214	98.2	
Alcohol consumption	Yes	113	51.8	131	60.1	0.004
	No	105	48.2	87	39.9	
Hours of sleep	<7 h/day	133	61.0	114	52.3	0.043
	≥7 h/day	85	39.0	104	47.7	
Physical activity	Low	85	39.0	62	28.4	0.020
	Medium	96	44.0	125	57.3	
	High	37	17.0	31	14.2	
Other job	Yes	40	18.3	41	18.8	0.808
	No	178	81.7	177	81.2	
Work shift	Duty	68	31.2	86	39.4	0.004
	Diurnal	146	67.0	129	59.2	
	Nocturnal	04	1.8	03	1.4	
Workday	≤40 h/week	68	31.2	48.0	22.0	0.006
	>40 h/week	150	68.8	170	78.0	
Occupational stress	High stress	31	14.2	61	27.9	<0.001
	Low stress	187	85.8	157	72.1	
Health considerations	Bad	13	6.0	08	3.7	0.394
	Very good	21	9.6	27	12.4	
	Good	112	51.4	103	47.2	
	Regular	72	33.0	80	36.7	
Positive diagnosis of COVID-19	Yes	-	-	66	30.3	-
	No	-	-	152	69.7	
**Continuous Variables**	**Before (2019)**		**During (2020)**			***p*-Value ##**
	**Mean (Standard Deviation)**		**Mean (Standard Deviation)**			
Age	32.58 (8.3)		33.59 (8.3)			<0.001
Weight	69.23 (14.0)		70.84 (14.4)			<0.001
BMI	25.52 (4.6)		26.11 (4.7)			<0.001
WC	84.25 (11.2)		87.69 (11.7)			<0.001
BF%	28.30 (7.5)		29.50 (7.3)			<0.001
FFM%	71.7 (7.4)		70.3 (7.2)			<0.001

BMI = body mass index; WC = waist circumference; BF% = body fat percentage; FFM = fat-free mass. * MW = Minimum wage for 2019 (USD 186.46). # McNemar chi-square test or Wilcoxon test. ## Student’s *t*-test.

**Table 3:** Distribution of factorial loads for the three food consumption patterns of hospital workers identified in the study. Santo Antônio de Jesus, Bahia, Brazil 2020.

Food Groups	Eating Patterns during the COVID-19 Pandemic		
	Pattern A	Pattern B	Pattern C
Breads and cereals	*	0.334	0.650
Tubers	0.619	*	*
Fruits	0.680	*	*
Vegetable	0.744	*	*
Legumes	*	*	*
Oilseeds	0.650	*	*
Eggs	0.519	*	*
Meat	*	0.607	*
Milk and dairy products	*	*	0.655
Fats	*	0.411	0.643
Pasta and other preparations	*	0.766	*
Candy	*	0.390	*
Drinks	*	−0.325	0.502
Sugary drinks	*	0.654	*
Variance (%)	17.20	14.71	12.56
Kaiser–Meyer–Olkin (KMO)	0.713		

* The food group was not included in the pattern studied.

**Table 4:** Structural equation modeling for the association between dietary patterns, occupational stressors and workers’ body composition. Santo Antônio de Jesus, Bahia, Brazil, 2020.

Direct Effect	Standardized Coefficients	*p*-Value	CI95%
Pattern A ⇒ stressors	−0.133	0.185	−0.086; 0.016
Pattern B ⇒ stressors	0.225	0.023	0.007; 0.108
Pattern C ⇒ stressors	0.114	0.278	−0.024; 0.083
Stressors ⇒ BMI	0.478	<0.001	−13.801; −3.657
Stressors ⇒ WC	0.395	0.001	−28.590; −6.948
Stressors ⇒ LM	−0.104	0.292	−10.685; 3.213
Stressors ⇒ FFM	0.440	0.001	−22.388; −5.434
**Indirect effect**			
Pattern A ⇒ stressors ⇒BMI	0.064	0.155	−0.114; 0.721
Pattern A ⇒ stressors ⇒ WC	0.053	0.166	−0.257; 1.492
Pattern A ⇒ stressors ⇒ LM	0.014	0.370	−0.154; 0.414
Pattern A ⇒ stressors ⇒ FFM	0.059	0.157	−0.186; 1.153
Pattern B ⇒ stressors ⇒ BMI	0.107	0.025	−0.953; −0.063
Pattern B ⇒ stressors ⇒ WC	0.089	0.027	−1.952; −0.115
Pattern B ⇒ stressors ⇒ LM	0.023	0.303	−0.631; 0.196
Pattern B ⇒ stressors ⇒ FFM	0.099	0.032	−1.549; −0.069
Pattern C ⇒ stressors ⇒ BMI	−0.054	0.268	−0.720; 0.200
Pattern C ⇒ stressors ⇒ WC	−0.045	0.273	−1.476; 0.417
Pattern C ⇒ stressors ⇒ LM	−0.012	0.429	−0.387; 0.164
Pattern C ⇒ stressors ⇒ FM	−0.050	0.270	−1.151; 0.322
**Fit indices**			
RMSEA	0.026		
SRMR	0.050		
TLI	0.978		
CFI	0.985		

BMI = body mass index; WC = waist circumference; FFM = fat-free mass; LM = lean mass; CI95% = 95% Confidence Intervals; RMSEA = Root Mean Square Error of Approximation; SRMR = Standardized Root Mean Square Residual; TLI = Tucker–Lewis Index; CFI = Comparative Fit Index.

## Data Availability

Not applicable.

## References

[1] Bendassoli P.F. (2012). Saúde e Trabalho Podem Caminhar Juntos?. Soc. E Gestão.

[2] Ros M., Schwartz S.H., Surkiss S. (1999). Basic Individual Values, Work Values, and the Meaning of Work. Int. Assoc. Appl. Psychol..

[3] Roe R.A., Ester P. (1999). Values and Work Empirical Findings and Theoretical Perspective. Appl. Psychol. Int. Rev..

[4] Désiron H.A.M., Donceel P., Godderis L., Van Hoof E., de Rijk A. (2015). What Is the Value of Occupational Therapy in Return to Work for Breast Cancer Patients? A Qualitative Inquiry among Experts. Eur. J. Cancer Care.

[5] Waddell G., Burton A.K., Great B. (2006). Department for Work and Pensions. Is Work Good for Your Health and Well-Being?.

[6] World Health Organization (1995). Global Strategy on Occupational Health for All.

[7] Takala E.P., Pehkonen I., Forsman M., Hansson G.Å., Mathiassen S.E., Neumann W.P., Sjøgaard G., Veiersted K.B., Westgaard R.H., Winkel J. (2010). Systematic Evaluation of Observational Methods Assessing Biomechanical Exposures at Work. Scand. J. Work. Environ. Health.

[8] Tawiah P.A., Baffour-Awuah A., Appiah-Brempong E., Afriyie-Gyawu E. (2022). Identifying Occupational Health Hazards among Healthcare Providers and Ancillary Staff in Ghana: A Scoping Review Protocol. BMJ Open.

[9] de Lima Santana L., Sarquis L.M.M., Miranda F.M.D.A., Kalinke L.P., Felli V.E.A., Mininel V.A. (2016). Health Indicators of Workers of the Hospital Area. Rev. Bras. De Enferm..

[10] Holmes C.J., Racette S.B. (2021). The Utility of Body Composition Assessment in Nutrition and Clinical Practice: An Overview of Current Methodology. Nutrients.

[11] Barreiro P.L.D., Vasconcelos A.G.G., Rotenberg L., Griep R.H., de Aguiar O.B. (2020). Dietary Patterns in a Nursing Team Measured by Principal Component Analysis. Rev. Da Esc. De Enferm..

[12] Betancourt-Nuñez A., Márquez-Sandoval F., González-Zapata L.I., Babio N., Vizmanos B. (2018). Unhealthy Dietary Patterns among Healthcare Professionals and Students in Mexico. BMC Public Health.

[13] Schulze M.B., Martínez-González M.A., Fung T.T., Lichtenstein A.H., Forouhi N.G. (2018). Food Based Dietary Patterns and Chronic Disease Prevention. BMJ.

[14] Kac G., Sichiery R., Gigante D.P. (2007). Epidemiologia Nutricional.

[15] Hosseinzadeh M., Vafa M., Esmaillzadeh A., Feizi A., Majdzadeh R., Afshar H., Keshteli A.H., Adibi P. (2016). Empirically Derived Dietary Patterns in Relation to Psychological Disorders. Public Health Nutr..

[16] Kunene S.H., Taukobong N.P. (2017). Dietary Habits among Health Professionals Working in a District Hospital in KwaZulu-Natal, South Africa. Afr. J. Prim. Health Care Fam. Med..

[17] Nuhu N., Brown C.A., Ainuson-Quampah J. (2020). Association between Caloric Intake and Work-Related Stress among Nurses in Two District Hospitals in Ghana. HSI J..

[18] Doğan Y.N., Doğan İ., Kiliç İ. (2021). The Perception of Health and the Change in Nutritional Habits of Healthcare Professionals during the COVID-19 Pandemic. Prog. Nutr..

[19] Von Dem Knesebeck O., Klein J., Frie K.G., Blum K., Siegrist J. (2010). Psychosoziale Arbeitsbelastungen Bei Chirurgisch Tätigen Krankenhausärzten: Ergebnisse Einer Bundesweiten Befragung. Dtsch. Arztebl..

[20] Karasek R. (2008). Low Social Control and Physiological Deregulation-the Stress-Disequilibrium Theory, towards a New Demand-Control Model. SJWEH Suppl..

[21] Bauer J., Groneberg D.A. (2013). Ärztlicher Disstress—Eine Untersuchung Baden-Württembergischer Ärztinnen Und Ärzte in Krankenhäusern. Dtsch. Med. Wochenschr..

[22] Siegrist J. (1996). Adverse Health Effects of High-Effort/Low-Reward Conditions.

[23] Banwat M.E., Haruna S.A., Vongdip N.G., Duru A.K., Afolaranmi T.O. (2018). Assessment of the Nutritional Knowledge, Eating Habits and Nutritional Statuses of Healthcare Workers in Jos, North-Central Nigeria. Res. J. Food Sci. Nutr..

[24] Sanghera J., Pattani N., Hashmi Y., Varley K.F., Cheruvu M.S., Bradley A., Burke J.R. (2020). The Impact of SARS-CoV-2 on the Mental Health of Healthcare Workers in a Hospital Setting—A Systematic Review. J. Occup. Health.

[25] Varli S.N., Bilici S. (2016). The Nutritional Status of Nurses Working Shifts: A Pilot Study in Turkey. Rev. De Nutr..

[26] Jung H., Dan H., Pang Y., Kim B., Jeong H., Lee J.E., Kim O. (2020). Association between Dietary Habits, Shift Work, and the Metabolic Syndrome: The Korea Nurses Health Study. Int. J. Environ. Res. Public Health.

[27] Shafi Z., Arif S., Nasir A. (2020). Association of Long Duty Hours and Unhealthy Dietary Habits among Nurses at Private and Public Sector in Karachi, Pakistan. J. Liaquat Univ. Med. Health Sci..

[28] Nishitani N., Sakakibara H., Akiyama I. (2009). Eating Behavior Related to Obesity and Job Stress in Male Japanese Workers. Nutrition.

[29] Buss J. (2012). Associations between Obesity and Stress and Shift Work among Nurses. Workplace Health Saf..

[30] Morera L.P., Marchiori G.N., Medrano L.A., Defagó M.D. (2019). Stress, Dietary Patterns and Cardiovascular Disease: A Mini-Review. Front. Neurosci..

[31] Zhang J., Lai S., Lyu Q., Zhang P., Yang D., Kong J., Qi Y., Yuan W., Zeng S., Song P. (2020). Diet and Nutrition of Healthcare Workers in COVID-19 Epidemic—Hubei, China, 2019. China CDC Wkly..

[32] Islam T., Ara I., Parvin R., Rozario M. (2021). Dietary Patterns of Health Workers in COVID Dedicated Hospital. Int. J. Nat. Soc. Sci..

[33] World Health Organization (2015). Guideline: Sugar Intake for Adults and Children.

[34] Bleier H., Lützerath J., Schaller A. (2022). Organizational Framework Conditions for Workplace Health Management in Different Settings of Nursing—A Cross-Sectional Analysis in Germany. Int. J. Environ. Res. Public Health.

[35] Negri Filho A.A. (2016). de Bases Para Um Debate Sobre a Reforma Hospitalar Do SUS: As Necessidades Sociais e o Dimensionamento e Tipologia de Leitos Hospitalares Em Um Contexto de Crise de Acesso e Qualidade.

[36] Eickemberg M., Cunha De Oliveira C., Karla A., Roriz C., Abreu G., Fontes V., Mello A.L., Sampaio R. (2013). Bioimpedância Elétrica e Gordura Visceral: Uma Comparação Com a Tomografia Computadorizada Em Adultos e Idosos Bioelectrical Impedance and Visceral Fat: A Comparison with Computed Tomography in Adults and Elderly. Arquivos Brasileiros de Endocrinologia Metabologia.

[37] Abashzadeh K., Siassi F., Qorbani M., Koohdani F., Farasati N., Sotoudeh G. (2017). The Study of Dietary Patterns and Their Relationship to Anthropometryin Female Nurses. Tehran Univ. J..

[38] Rosner B. (2010). Fundamentals of Biostatistics.

[39] Coelho L., Costa P., Kinra S., Mallinson P., Akutsu R. (2023). Association between occupational stress, work shift and health outcomes in hospital workers of the Recôncavo of Bahia, Brazil: The impact of COVID-19 pandemic. Br. J. Nutr..

[40] Craig C.L., Marshall A.L., Sjöström M., Bauman A.E., Booth M.L., Ainsworth B.E., Pratt M., Ekelund U., Yngve A., Sallis J.F. (2003). International Physical Activity Questionnaire: 12-Country Reliability and Validity. Med. Sci. Sport. Exerc..

[41] World Health Organization (1995). El Estado Físico: Uso e Interpretación de La Antropometría.

[42] World Health Organization (2000). Obesity: Preventing and Managing the Global Epidemic.

[43] World Health Organization (2008). Waist Circumference and Waist-Hip Ratio: Report of a WHO Expert Consultation.

[44] Kyle U.G., Bosaeus I., De Lorenzo A.D., Deurenberg P., Elia M., Gómez J.M., Heitmann B.L., Kent-Smith L., Melchior J.C., Pirlich M. (2004). Bioelectrical Impedance Analysis—Part II: Utilization in Clinical Practice. Clin. Nutr..

[45] Guedes D.P. (1998). Controle Do Peso Corporal: Composição Corporal, Atividade Física e Nutrição.

[46] del Carmen Bisi Molina M., Benseñor I.M., de Oliveira Cardoso L., Velasquez-Melendez G., Drehmer M., Sabrina Silva Pereira T., Perim de Faria C., Melere C., Manato L., Lizabeth Costa Gomes A. (2013). Reprodutibilidade e Validade Relativa Do Questionário de Frequência Alimentar Do ELSA-Brasil. Cad. De Saúde Pública.

[47] Guimarães M., Alves M., Chor D., Faerstein E., De C., Lopes C.E., Guilherme S., Werneck L. (2004). A Portuguese-Language Adaptation. Adaptação da Job Stress Scale Rev Saúde Pública.

[48] Keller E., Widestrom M., Gould J., Fang R., Davis K.G., Gillespie G.L. (2022). Examining the Impact of Stressors during COVID-19 on Emergency Department Healthcare Workers: An International Perspective. Int. J. Environ. Res. Public Health.

[49] Hair J.F., Black W.C., Babin B.J., Anderson R.E., Tatham R.L. (2009). Análise Multivariada de Dados.

[50] de Arruda Neta A.d.C.P., Steluti J., de Lima Ferreira F.E.L., de Farias Junior J.C., Marchioni D.M.L. (2021). Dietary Patterns among Adolescents and Associated Factors: Longitudinal Study on Sedentary Behavior, Physical Activity, Diet and Adolescent Health. Cienc. E Saude Coletiva.

[51] Marôco J. (2014). Análise De Equações Estruturais-Fundamentos Teóricos.

[52] Li C.-H. (2016). The Performance of ML, DWLS, and ULS Estimation With Robust Corrections in Structural Equation Models With Ordinal Variables. Psychol. Methods.

[53] Maccallum R.C., Browne M.W., Sugawara H.M. (1996). Power Analysis and Determination of Sample Size for Covariance Structure Modeling. Psychol. Methods.

[54] Bentler P.M. (1990). Comparative Fit Indexes in Structural Models. Psychol. Bull..

[55] Nobre L.N., Monteiro J.B.R. (2003). Determinantes Dietéticos Da Ingestão Alimentar e Efeito Na Regulação Do Peso Corporal. Arch. Latinoam. De Nutr..

[56] Associação Brasileira para o Estudo da Obesidade e da Síndrome Metabólica (2021). Diretrizes Brasileiras de Obesidade 2016.

[57] Bell E.A., Rolls B.J. (2001). Energy Density of Foods Affects Energy Intake across Multiple Levels of Fat Content in Lean and Obese Women. Am. J. Clin. Nutr..

[58] Brotman D.J. (2007). Sympathetic Nervous System The Cardiovascular Toll of Stress. Lancet.

[59] Solovieva S., Lallukka T., Virtanen M., Viikari-Juntura E. (2013). Psychosocial Factors at Work, Long Work Hours, and Obesity: A Systematic Review. Scand. J. Work. Environ. Health.

[60] Kivimäki M., Singh-Manoux A., Nyberg S., Jokela M., Virtanen M. (2015). Job Strain and Risk of Obesity: Systematic Review and Meta-Analysis of Cohort Studies. Int. J. Obes..

[61] Reiter R.J., Tan D.X., Korkmaz A., Ma S. (2012). Obesity and Metabolic Syndrome: Association with Chronodisruption, Sleep Deprivation, and Melatonin Suppression. Ann. Med..

[62] Peplonska B., Bukowska A., Sobala W. (2015). Association of Rotating Night Shift Work with BMI and Abdominal Obesity among Nurses and Midwives. PLoS ONE.

[63] Khosravipour M., Shahmohammadi M., Athar H.V. (2019). The Effects of Rotating and Extended Night Shift Work on the Prevalence of Metabolic Syndrome and Its Components. Diabetes Metab. Syndr. Clin. Res. Rev..

[64] Almajwal A.M. (2016). Stress, Shift Duty, and Eating Behavior among Nurses in Central Saudi Arabia. Saudi Med. J..

[65] Han K., Trinkoff A.M., Geiger-Brown J. (2014). Factors Associated with Work-Related Fatigue and Recovery in Hospital Nurses Working 12-Hour Shifts. Workplace Health Saf..

[66] Bonham M.P., Bonnell E.K., Huggins C.E. (2016). Energy Intake of Shift Workers Compared to Fixed Day Workers: A Systematic Review and Meta-Analysis. Chronobiol. Int..

[67] Bello G.B., Silva F.M., Dier C., Schneider A.P. (2015). Associação Entre o Índice Glicêmico e a Carga Glicêmica Da Dieta Atual de Frequentadores de Clínicas Estéticas Privadas de Porto Alegre—RS e Indicadores de Adiposidade Corporal. Nutrire.

[68] Clar C., Al-Khudairy L., Loveman E., Kelly S.A.M., Hartley L., Flowers N., Germanò R., Frost G., Rees K. (2017). Low Glycaemic Index Diets for the Prevention of Cardiovascular Disease. Cochrane Database Syst. Rev..

[69] Mohammed T., Hazmi A., Alghamdi A., Abdulmajeed I. (2018). Eating Habits among Healthcare Providers during Working Hours at National Guard Health Affairs-Riyadh, Saudi Arabia. Int. J. Med. Res. Health Sci..

[70] De Oliveira Izar M.C., Lottenberg A.M., Giraldez V.Z.R., Dos Santos Filho R.D., Machado R.M., Bertolami A., Assad M.H.V., Saraiva J.F.K., Faludi A.A., Moreira A.S.B. (2021). Position Statement on Fat Consumption and Cardiovascular Health-2021. Arq. Bras. Cardiol..

[71] Sun Y., Neelakantan N., Wu Y., Lote-Oke R., Pan A., van Dam R.M. (2015). Palm Oil Consumption Increases LDL Cholesterol Compared with Vegetable Oils Low in Saturated Fat in a Meta-Analysis of Clinical Trials. J. Nutr..

[72] Tholstrup T., Hjerpsted J., Raff M. (2011). Palm Olein Increases Plasma Cholesterol Moderately Compared with Olive Oil in Healthy Individuals. Am. J. Clin. Nutr..

[73] Chung M.G., Li Y., Liu J. (2021). Global Red and Processed Meat Trade and Non-Communicable Diseases. BMJ Glob. Health.

[74] Rouhani M.H., Salehi-Abargouei A., Surkan P.J., Azadbakht L. (2014). Is There a Relationship between Red or Processed Meat Intake and Obesity?. A Systematic Review and Meta-Analysis of Observational Studies. Obes. Rev..

[75] de Vasconcelos Ramos A.P.L., Pereira L.Z., Neto M.T.R., de Rezende F.V. (2021). Occupational Stress in Managers of a Public Hospital. Interacao Em Psicol..

[76] Dobson K.G., Gilbert-Ouimet M., Mustard C., Smith P.M. (2020). Body Mass Index Trajectories among the Canadian Workforce and Their Association with Work Environment Trajectories over 17 Years. Occup. Environ. Med..

[77] Berset M., Semmer N.K., Elfering A., Jacobshagen N., Meier L.L. (2011). Does Stress at Work Make You Gain Weight? A Two-Year Longitudinal Study. Scand. J. Work. Environ. Health.

[78] Fujishiro K., Lawson C.C., Hibert E.L., Chavarro J.E., Rich-Edwards J.W. (2015). Job Strain and Changes in the Body Mass Index among Working Women: A Prospective Study. Int. J. Obes..

[79] Toyoshima H., Masuoka N., Hashimoto S., Otsuka R., Sasaki S., Tamakoshi K., Yatsuya H. (2009). Effect of the Interaction between Mental Stress and Eating Pattern on Body Mass Index Gain in Healthy Japanese Male Workers. J. Epidemiol..

[80] Takaki J., Minoura A., Irimajiri H., Hayama A., Hibino Y., Kanbara S., Sakano N., Ogino K. (2010). Interactive Effects of Job Stress and Body Mass Index on Over-Eating. J. Occup. Health.

[81] Hemiö K., Lindström J., Peltonen M., Härmä M., Viitasalo K., Puttonen S. (2020). The Association of Work Stress and Night Work with Nutrient Intake—A Prospective Cohort Study. Scand. J. Work. Environ. Health.

[82] Würtz A.M., Kinnerup M.B., Pugdahl K., Schlünssen V., Vester-Gaard J.M., Nielsen K., Cramer C., Bonde J.P., Biering K., Carstensen O. (2022). Healthcare Workers’ SARS-CoV-2 Infection Rates during the Second Wave of the Pandemic: Follow-up Study. Scand. J. Work. Environ. Health.

[83] Chinvararak C., Kerdcharoen N., Pruttithavorn W., Polruamngern N., Asawaroekwisoot T., Munsukpol W., Kirdchok P. (2022). Mental Health among Healthcare Workers during COVID-19 Pandemic in Thailand. PLoS ONE.

[84] Mota I.A., De Oliveira Sobrinho G.D., Morais L.P.S., Dantas T.F. (2021). Impact of COVID-19 on Eating Habits, Physical Activity and Sleep in Brazilian Healthcare Professionals. Arq. De Neuro-Psiquiatr..

[85] Jackson C.L., Wee C.C., Hurtado D.A., Kawachi I. (2016). Obesity Trends by Industry of Employment in the United States, 2004 to 2011. BMC Obes..

[86] Yamada Y., Kameda M., Noborisaka Y., Suzuki H., Honda M., Yamada S. (2001). Excessive Fatigue and Weight Gain among Cleanroom Workers after Changing from an 8-Hour to a 12-Hour Shift. Scand. J. Work. Environ. Health.

[87] Niedhammer I., Lert F., Marne M.J. (1996). Prevalence of Overweight and Weight Gain in Relation to Night Work in a Nurses’ Cohort. Int. J. Obes. Relat. Metab. Disord..

[88] Di Lorenzo L., De Pergola G., Zocchetti C., L’Abbate N., Basso A., Pannacciulli N., Cignarelli M., Giorgino R., Soleo L. (2003). Effect of Shift Work on Body Mass Index: Results of a Study Performed in 319 Glucose-Tolerant Men Working in a Southern Italian Industry. Int. J. Obes..

[89] Drewnowski A., Specter S. (2004). Poverty and Obesity: The Role of Energy Density and Energy Costs. Am. J. Clin. Nutr..

